# Subgingival microbiota dysbiosis in systemic lupus erythematosus: association with periodontal status

**DOI:** 10.1186/s40168-017-0252-z

**Published:** 2017-03-20

**Authors:** Jôice Dias Corrêa, Débora Cerqueira Calderaro, Gilda Aparecida Ferreira, Santuza Maria Souza Mendonça, Gabriel R. Fernandes, E. Xiao, Antônio Lúcio Teixeira, Eugene J. Leys, Dana T. Graves, Tarcília Aparecida Silva

**Affiliations:** 1 Faculty of Dentistry, Universidade Federal de Minas Gerais, Belo Horizonte, Minas Gerais Brazil; 2 University Hospital, Universidade Federal de Minas Gerais, Belo Horizonte, Minas Gerais Brazil; 3 René Rachou Research Center, Oswaldo Cruz Foundation, Belo Horizonte, Minas Gerais Brazil; 4 Penn Dental School, University of Pennsylvania, Philadelphia, PA USA; 5 College of Dentistry, The Ohio State University, Columbus, OH USA; 6 Departamento de Patologia e Cirurgia Odontológica, Faculdade de Odontologia, Universidade Federal de Minas Gerais, Av. Antônio Carlos 6627, CEP 31.270-901 Belo Horizonte, Minas Gerais Brazil

**Keywords:** Lupus, Periodontitis, Oral microbiota, Cytokine, Subgingival dental plaque, Illumina sequencing

## Abstract

**Background:**

Periodontitis results from the interaction between a subgingival biofilm and host immune response. Changes in biofilm composition are thought to disrupt homeostasis between the host and subgingival bacteria resulting in periodontal damage. Chronic systemic inflammatory disorders have been shown to affect the subgingival microbiota and clinical periodontal status. However, this relationship has not been examined in subjects with systemic lupus erythematosus (SLE). The objective of our study was to investigate the influence of SLE on the subgingival microbiota and its connection with periodontal disease and SLE activity.

**Methods:**

We evaluated 52 patients with SLE compared to 52 subjects without SLE (control group). Subjects were classified as without periodontitis and with periodontitis. Oral microbiota composition was assessed by amplifying the V4 region of 16S rRNA gene from subgingival dental plaque DNA extracts. These amplicons were examined by Illumina MiSeq sequencing.

**Results:**

SLE patients exhibited higher prevalence of periodontitis which occurred at a younger age compared to subjects of the control group. More severe forms of periodontitis were found in SLE subjects that had higher bacterial loads and decreased microbial diversity. Bacterial species frequently detected in periodontal disease were observed in higher proportions in SLE patients, even in periodontal healthy sites such as *Fretibacterium*, *Prevotella nigrescens*, and *Selenomonas*. Changes in the oral microbiota were linked to increased local inflammation, as demonstrated by higher concentrations of IL-6, IL-17, and IL-33 in SLE patients with periodontitis.

**Conclusions:**

SLE is associated with differences in the composition of the microbiota, independently of periodontal status.

**Electronic supplementary material:**

The online version of this article (doi:10.1186/s40168-017-0252-z) contains supplementary material, which is available to authorized users.

## Background

Periodontitis is a multifactorial infectious disease that affects tooth supporting tissues [1]. In health, there is equilibrium between the subgingival biofilm and the host immune response. Periodontal bone loss is initiated when there is a disruption that causes the inflammatory response to move closer to the bone inducing osteoclastogenesis and impaired osseous coupling [2]. Changes in bacterial composition or systemic inflammation are thought to disrupt the balance between host and oral microbiota leading to destruction of periodontal tissues [3–5]. Therefore, chronic inflammatory conditions such as diabetes, obesity, rheumatoid arthritis (RA), and systemic lupus erythematosus (SLE) are linked to an increased risk or severity of periodontal disease [6–11]. Furthermore, periodontitis might have an adverse effect by enhancing systemic inflammation and increasing the risk of myocardial infarction [12], preterm birth [7], pneumonia [13], stroke [14], and rheumatoid arthritis [15] as well as worsen SLE severity [16].

SLE is an autoimmune disease characterized by the presence of aberrant antibody responses to nuclear and cytoplasmic antigens. It is a complex multifactorial disease in which genetic and environmental factors contribute to disease susceptibility [17]. An environmental factor that modulates autoimmunity is the bidirectional crosstalk between the human host and microbiota, as reported for rheumatic diseases [18, 19]. However, few studies have addressed the relationship between SLE and the human microbiota [20, 21] or the periodontal condition in SLE patients [10, 16, 22–27]. Moreover, the results have been conflicting, due in part to small sample size.

The influence of SLE on the oral microbiota has not been reported. To address this issue, we analyzed the subgingival microbiota in SLE patients and healthy subjects to draw potential links among systemic immune dysregulation, subgingival microbiota, and periodontal disease. The results indicate that SLE alters the subgingival microbiota. This understanding might be useful in delineating strategies to treat these patients and suggests that these patients should be followed closely for periodontal disease based on their altered microbiota and enhanced susceptibly to develop periodontitis.

## Results

### Systemic lupus erythematosus results in higher prevalence and severity of periodontal disease

Demographic and clinical characteristics of subjects included in the study are presented in Table 1. Sixty-seven percent of SLE patients had periodontitis, a significantly higher prevalence when compared to healthy controls (53%). The prevalence of chronic periodontitis in healthy Brazilians is consistent with previous reports [28–30]. An interesting finding was the presence of periodontitis at a younger age in SLE patients (40.5 ± 10.1 years) compared to controls (46.3 ± 13.2, *p* < 0.05). In addition, 29 SLE patients were younger than 35 years, and from these, 52% already had periodontitis (data not shown). SLE subjects had increased probing depth and increased loss of clinical attachment indicating more severe periodontitis (Table 1). Periodontal probing depth was significantly correlated with most of the SLE parameters (duration of SLE rho = 0.32, accumulated dose of prednisone rho = 0.36 and C-reactive protein level rho = 0.30, *p* < 0.05). Moreover, loss of clinical attachment was correlated with duration of SLE (rho = 0.35, *p* < 0.05) and the Systemic Lupus International Collaborating Clinics/American College of Rheumatology Damage Index (SLICC/ACR) damage index (rho = 0.32, *p* < 0.05).

**Table 1:** Demographic and clinical data of patients with SLE and healthy control subjects

	Controls		SLE	
	Non-CP	CP	Non-CP	CP
Subjects	24 (46.2%)	28 (53.8%)	17 (32.7%)	**35 (67.3%)** ^ab^
Females (%)	39	48	30	57
Age (years)	34.7 (±15.9)	46.3 (±13.2)^a^	31.2 (±5.2)	**40.5 (±10.1)** ^ab^
Smoking status (%)	3.81	7.60	5.70	9.60
SLE duration (years)	–	–	8.6 (±6.2)	12.5 (±7.8)^a^
SLEDAI	–	–	4.5 (±4.0)	5.5 (±4.6)
SLICC/ACR	–	–	0.5 (±1.0)	0.9 (±0.9)
Probing depth (mm)	1.65 (±0.31)	2.72 (±0.62)^a^	1.91 (±0.62)	**3.37 (±0.49)** ^ab^
Clinical attachment level (mm)	2.60 (±0.24)	3.62 (±0.63)^a^	2.97 (±0.63)	**4.00 (±0.63)** ^ab^
Bleeding upon probing (% sites)	4.67 (±7.85)	20.78(±19.5)^a^	7.20 (±4.70)	16.91 (±15.9)^a^
Plaque Index (score)	0.43 (±0.28)	0.91(±0.59)^a^	0.83 (±0.40)	0.88 (±0.50)
Tooth brushing (times/day)	2.85 (±0.93)	2.62 (±0.85)	3.0 (±0.76)	2.96 (±0.92)
Dental floss (times/day)	1.06 (±0.94)	0.74 (±0.89)	1.26 (±1.40)	1.38 (±1.30)
Tooth loss	1.78 (±2.17)	4.50 (±3.70)^a^	1.38 (±1.57)	4.90 (±4.17)^a^

Values were expressed as mean ± SD or percentage

*Non-CP* without chronic periodontitis, *CP* chronic periodontitis, *SLEDAI* Systemic Lupus Erythematosus Disease Activity Index, *SLICC/ACR* Systemic Lupus International Collaborating Clinics/American College of Rheumatology Damage Index

^a^Statistically different comparing Non-CP × CP within the same group

^b^Statistically different comparing SLE × healthy control group. Kruskal-Wallis test with Dunn’s post hoc test <0.05

All subjects exhibited similar oral hygiene habits (frequency of tooth brushing and dental flossing per day). No difference in smoking status between groups was found. When evaluating oral hygiene parameters, control subjects with periodontitis had an increased plaque index compared to non-periodontitis subjects (*p* < 0.05), but this association was not observed in SLE patients (Table 1). It is noteworthy that when 336 SLE patients were evaluated, 62 (24%) were excluded because they did not have at least 8 teeth. Although it is difficult to assess the exact cause of this early tooth loss, many patients reported that they lost their teeth because the teeth became “loose,” consistent with loss from periodontitis.

### SLE impacts oral microbiota diversity

**Fig. 1:**
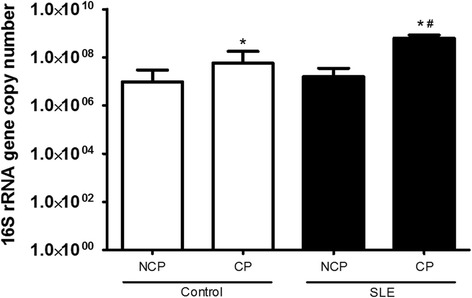
Subgingival bacterial load of healthy subjects (control) and patients with Systemic Lupus Erythematosus (SLE) with chronic periodontitis (CP) and Non-CP (NCP), determined by real-time PCR using universal primers for 16S rRNA gene. *Statistically different compared to NCP subjects within the same group. #Statistically different compared to control subjects. *p* < 0.05, Kruskal-Wallis test with Dunn’s post hoc test

**Fig. 2:**
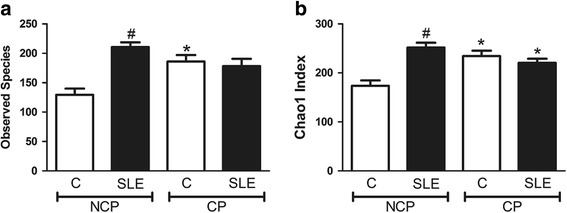
Alpha diversity index, **a** observed species and **b** Chao1 index of control subjects or Systemic Lupus Erythematosus patients (SLE) with chronic periodontitis (CP) or Non-CP (NCP). *Statistically different compared to NCP subjects within the same group. #Statistically different compared to Control group. *p* < 0.05, Kruskal-Wallis test with Dunn’s post hoc test. Alpha diversity metrics were calculated after subsampling to obtain equal number of sequences per library

To assess the factors that may lead to increased prevalence and severity of periodontitis in SLE patients, we examined whether the subgingival microbiota was altered. The bacterial load was one log higher in SLE diseased sites compared to control subjects with diseased sites (Fig. 1). In control subjects, the presence of periodontitis resulted in increased microbial diversity (*p* < 0.05), while in SLE patients, there was decreased microbial diversity at diseased sites (*p* < 0.05) (Fig. 2a and b).

**Fig. 3:**
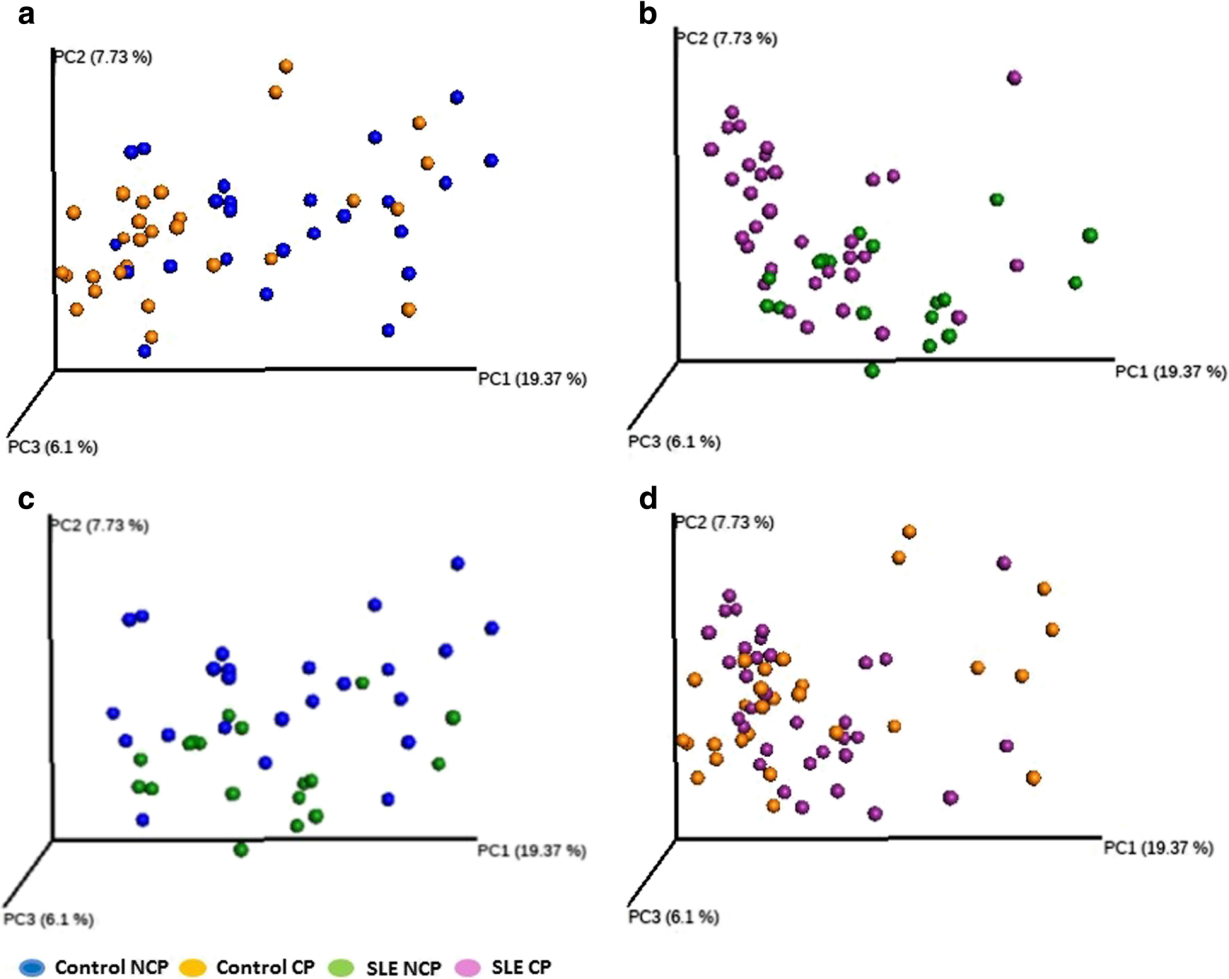
Beta diversity. Microbiota separation on the principal coordinates calculated from unweighted UniFrac distances. **a** Control subjects, **b** SLE patients, **c** non-periodontitis subjects, and **d** chronic periodontitis subjects

**Fig. 4:**
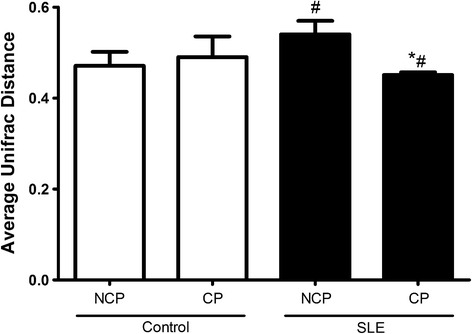
The average weighted UniFrac distance values (the beta diversities) of healthy subjects (control) and patients with Systemic Lupus Erythematosus (SLE) with chronic periodontitis (CP) and Non-CP (NCP).*Statistically different compared to NCP subjects within the same group. #Statistically different compared to control group. *p* < 0.05, Kruskal-Wallis test with Dunn’s post hoc test

UniFrac analysis was performed to compare the degree of phylogenetic overlap in the microbial communities in SLE and control subjects. Beta diversity, which reflects the relatedness of disease- and health-associated bacterial populations, established that bacterial communities from each group tended to cluster together (PERMANOVA, *p* = 0.001) (Fig. 3). The bacterial taxa in SLE diseased sites were more similar to each other than bacteria from SLE non-periodontitis and healthy subjects’ sites, indicating lower heterogeneity in subgingival microbial communities in the SLE periodontitis subjects (Fig. 4).

### Dysbiosis of oral microbiota in patients with SLE

**Fig. 5:**
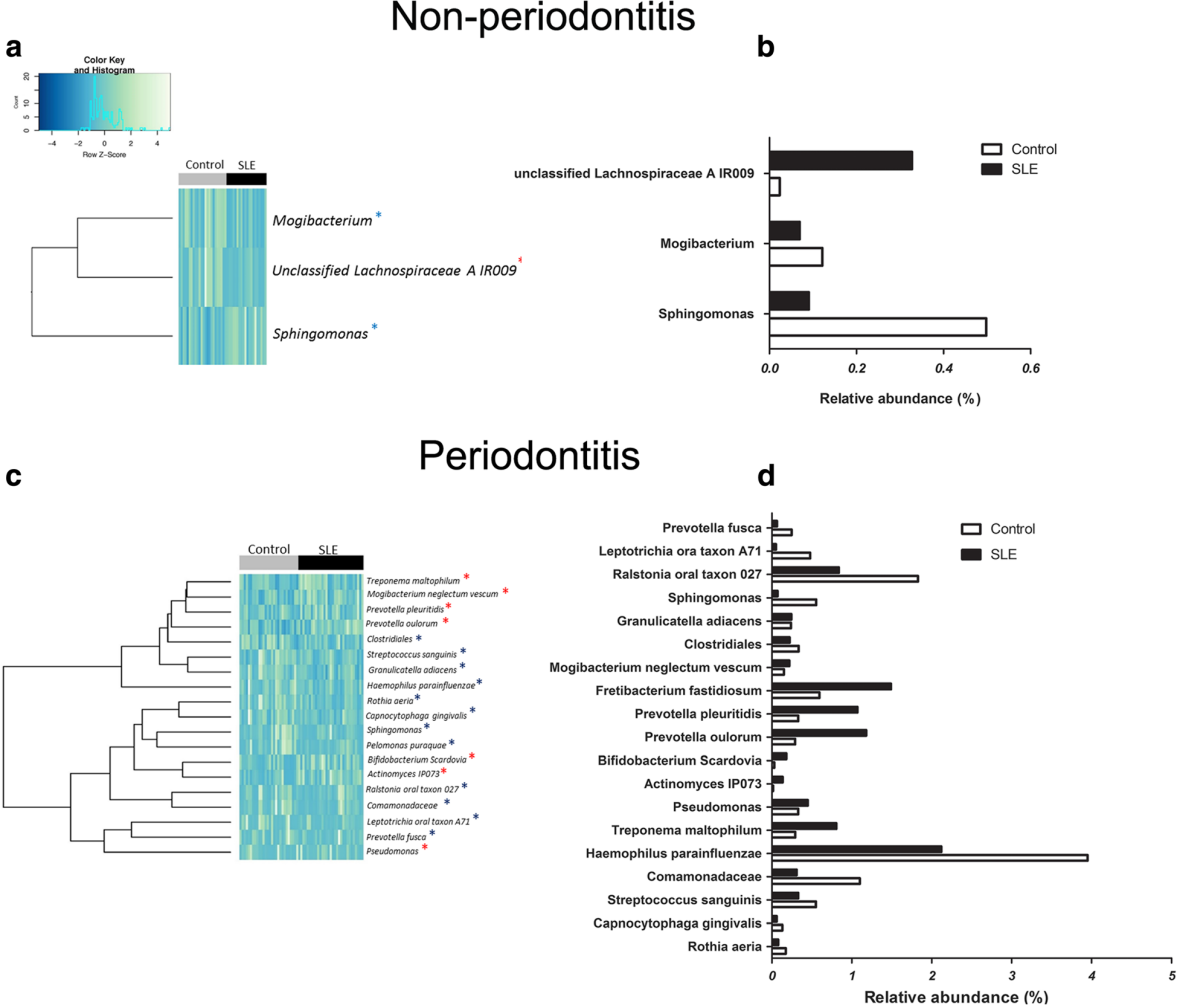
OTUs and taxa differing between non-periodontitis (*upper panel*) and periodontitis (*lower anel*) sites. **a**, **c** Heat map with the OTUs showing a *p* value of less than 0.05 when comparing relative abundances between control and SLE subjects. Subjects are shown in *columns*, while OTUs appear in *rows. Blue asterisks* indicate OTUs with decreased proportions in SLE patients, while *red asterisks* indicated OTUs with increased proportions in SLE in relation to respective controls. **b**, **d** The graphic representation of differences in the relative abundances of species represented in the heat maps

We investigated the impact of SLE on the subgingival bacterial composition. At healthy periodontal sites in SLE patients, there were increased proportions of a member of *Lachnospiraceae* family (unclassified *Lachnospiraceae* A IR009) compared to healthy sites in controls (*p* < 0.05) (Fig. 5a and b). In contrast, proportions of *Prevotella oulorum* and *Prevotella pleuritidis*, *Pseudomonas* spp, *Treponema maltophilum*, and *Actinomyces* IP073, bacteria associated with disease, were elevated at periodontal disease sites in patients with SLE compared to periodontitis sites of non-SLE controls (*p* < 0.05) (Fig. 5c and d). *Sphingomonas* were at lower relative levels in healthy periodontal sites of SLE patients (*p* < 0.05) (Fig. 5a and b) and *Rothia aeria*, *Capnocytophaga gingivalis*, *Clostridiales*, *Rasltonia* oral taxon 027, *Leptotrichia* oral taxon A71, *Streptococcus sanguinis*, and *Haemophilus parainfluenzae* had decreased relative abundance in periodontitis sites of SLE group (*p* < 0.05) (Fig. 5c and d)

**Fig. 6:**
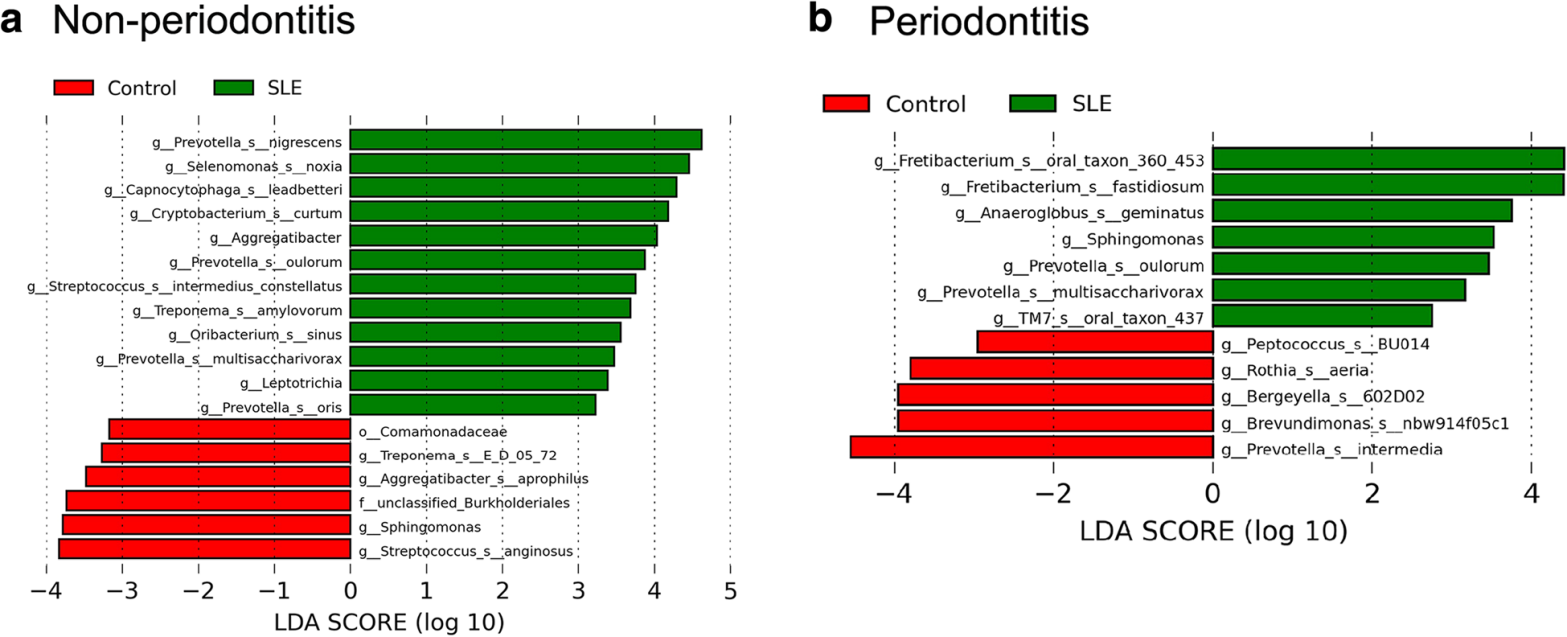
OTUs with different relative abundance in control subjects (*red*) and SLE samples (*green*) in non-periodontitis (**a**) and chronic periodontitis subjects (**b**). Graph depicts OTUs with different relative abundance based on LEfSe results. *Bars* represent linear discriminant analysis scores (LDA)

Differentially represented OTUs were analyzed via LEfSe, a statistical measure used in metagenomic biomarker discovery [31] (Fig. 6). This analysis revealed that at SLE healthy periodontal sites, species such as *Prevotella nigrescens*, *Prevotella oulorum*, *Prevotella oris*, and *Selenomonas noxia* exhibited elevated proportions (Fig. 6a). When examining the periodontitis sites, we found again increased relative levels of *P. oulorum* in SLE patients as well as increased relative levels of *Fretibacterium fastidiosum* and *Fusobacterium* oral taxon 360 450, *Anaeroglobus geminatus*, and *TM7* oral taxon 437 (Fig. 6b). Most of the bacterial species found with elevated proportions in SLE patients were anaerobic (*Prevotella*, *Selenomonas*, and *Treponema*), while in healthy non-SLE subjects, there was an increase in the relative abundance of predominantly aerobic species (*Rothia*, *Haemophilus*, and *Streptococcus*).

### Microbial intercorrelations

**Fig. 7:**
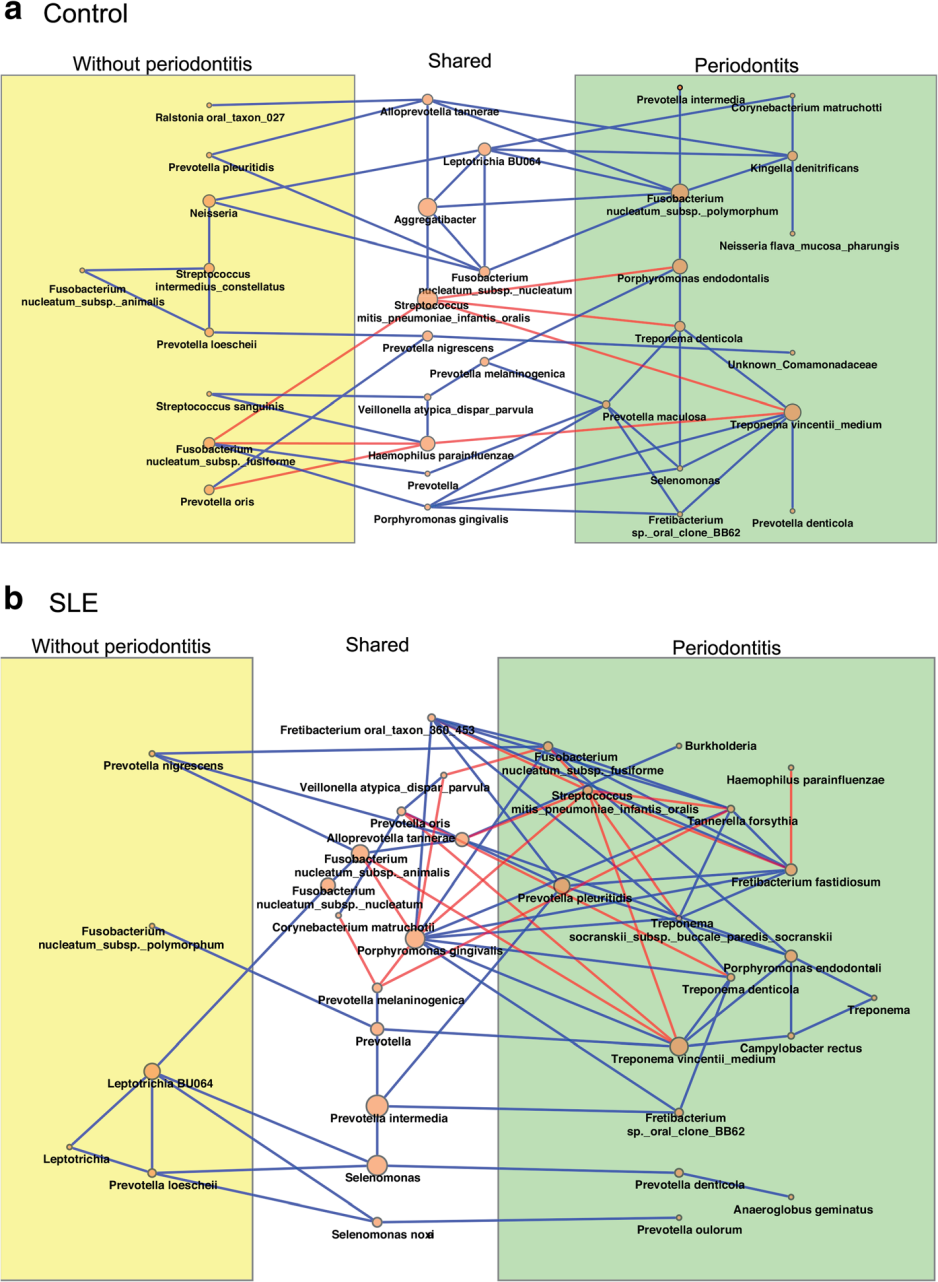
Correlations among bacteria species in control (**a**) and SLE subjects (**b**) with chronic periodontitis (CP) (*green*) and Non-CP (NCP) (*yellow*). In *white area* are the bacteria that are common to subjects with CP and NCP. Spearman Rank test with a cutoff rho value of 0.4 and *p* < 0.05. *Blue lines* indicate positive correlations and *red lines* negative correlations

Since periodontitis is a polymicrobial disease, it is important to investigate correlations among subgingival microorganisms. Fig. 7 shows the co-occurrence patterns among bacterial species in subjects with and without SLE. In non-SLE subjects (Fig. 7a), we observed a total of 89 bacterial correlations with positive relationship among pathogenic groups (i.e., *Porphyromonas gingivalis* and *Selenomonas*, *Treponema vincentii*, *Fusobacterium nucleatum*, and *Fretibacterium*) and negative correlations between pathogenic bacteria and periodontal healthy-related species (i.e., *Streptococcus mitis* and *Treponema denticola* and *T. vincentii* or between *P. oris* and *Haemophilus parainfluenzae*).

In SLE patients, we observed a wider net of correlations than in the healthy subjects, with a total of 111 correlations (Fig. 7b). Positive correlations were found among periodontal pathogens (*T. denticola*, *P. gingivalis*, *Fretibacterium fastidiosum*, *and Tannerella forsythia)* [32]. In contrast, negative correlations were observed among pathogenic bacteria (*F. fastidiosum*, *P. gingivalis*, *T. forsythia*, *T. denticola*, and *Prevotella intermedia*) and health-related species (*H. parainfluenzae* and *S. mitis*) in SLE group.

### Microbiota correlations with SLE index, salivary, and serum concentration of cytokines

**Fig. 8:**
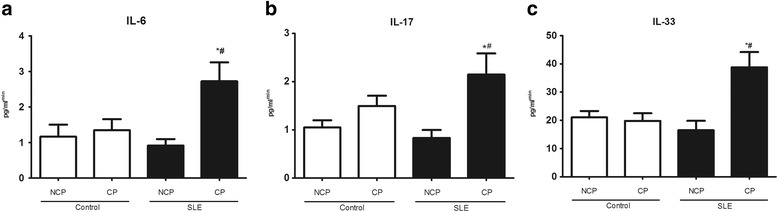
Concentration of IL-6 (**a**), IL-17 (**b**), and IL-33 (**c**) in saliva of healthy subjects (control) and patients with Systemic Lupus Erythematosus (SLE) with chronic periodontitis (CP) and Non-CP (NCP). Cytokine measurement was determined by CBA and ELISA, and values were normalized by stimulated salivary flux.*Statistically different compared to NCP subjects within the same group. #Statistically different compared to healthy subjects with CP. *p* < 0.05, Kruskal-Wallis test with Dunn’s post hoc test

We investigated the relationship between bacteria and local inflammation by measuring cytokines in saliva. There was a significant increase in inflammatory cytokines IL-6, IL-17, and IL-33 in saliva of SLE patients with periodontitis compared to control group with periodontitis. No significant differences in cytokine levels were found in non-SLE subjects with periodontitis compared to non-SLE subjects without periodontitis (Fig. 8). The production of these cytokines in saliva of SLE patients was correlated with relative abundance of *Selenomonas* (IL-6 rho = 0.42, IL-33 rho = 0.44, *p* < 0.05) *Prevotella denticola* (IL-33 rho = 0.40, *p* < 0.05), *Veilonella atypica* (IL-33 rho = 0.50, *p* < 0.05), and *Leptotrichia* (IL-17 rho = 0.4, *p* < 0.05). *Leptotrichia* was also correlated with IL-17 in healthy periodontal sites from SLE patients (rho = 0.7, *p* < 0.05). In the serum, there was a significant increase in the levels of IL-6 in the SLE patients with periodontitis, while no difference was found in other analyzed cytokines TNF-α, IL-33, and IFN-γ (Additional file 1: Table S1). No correlations were observed between IL-6 and relative abundance of bacteria.

Spearman Rank correlations were calculated to establish relationships between the relative abundance of bacterial taxa found in subgingival sites of SLE patients and parameters of SLE severity. Table 2 shows that six species of *Prevotella* correlated with SLE disease index and inflammatory markers. *Fretibacterium* oral taxon BB62 and *F. fastidiosum* correlated with SLE duration, while *S. noxia* correlated with CRP levels. *P. gingivalis* was positively correlated with SLE duration and accumulated dose of prednisone.

**Table 2:** Correlations among SLE parameters and relative abundance of bacteria species in subjects with chronic periodontitis (rho values)

	SLICC	SLEDAI	SLE duration (years)	Accumulated dose of prednisone (mg)	CRP	Neutrophils	Lymphocytes
					(mg/L)	(cells/mm^3^)	(cells/mm^3^)
*Prevotella nigrescens*	0.56*	0.40*	–	–	–	–	–
*Prevotella oris*	–	–	0.45*	–	–	–	–
*Prevotella denticola*	–	–	–	–	–	0.44*	–
*Prevotella melaninogenica*	–	–	–	–	0.44*	–	–
*Prevotella oulorum*	–	–	–	–	–	–	0.56*
*Fretibacterium oral taxon BB62*	–	–	0.51*	0.75*	–	–	–
*Fretibacterium fastidium*	–	–	0.40*	–	–	–	–
*Selenomonas noxia*	–	–	–	–	0.50*	–	–
*Porphyromonas gingivalis*	–	–	0.68*	0.48*	–	–	–

*SLEDAI* Systemic Lupus Erythematosus Disease Activity Index, *SLICC/ACR* Systemic Lupus International Collaborating Clinics/American College of Rheumatology Damage Index, *CRP* C-reactive protein

*Spearman rank correlation, *p* < 0.05

## Discussion

The major findings of this study are that SLE patients had a dysbiotic subgingival microbiota with higher subgingival bacterial load, reduced microbial diversity, and changes in bacterial composition with a shift toward greater proportions of pathogenic bacteria. The results suggest that SLE has a significant impact on periodontal health as SLE patients have more severe periodontitis. Reinforcing this finding, we also noticed that 24% of the initial SLE patients evaluated were excluded because they did not have at least eight teeth. In comparison, none of the healthy subjects were excluded for this reason. This finding is likely to be important since periodontal disease is an important cause of tooth loss in adults [33]. Moreover, periodontal probing depth, a measure of periodontal destruction, was correlated with the duration of SLE, accumulated dose of prednisone and the level of systemic inflammation measured by serum C-reactive protein.

We found a high prevalence of periodontitis in SLE patients, which was observed in almost 70% of these patients, in agreement with previous studies [16, 26, 34]. In our study, the experimental and control groups were similar regarding age and gender, ruling out these potential confounding factors [30]. Other factors may also contribute to the premature development of periodontal disease in SLE subjects, such as medications used to treat SLE. However, most are anti-inflammatory drugs consisting of steroids and immunosuppressive medications [35] that typically reduce gingival inflammation [36, 37]. We found that the dose of prednisone was correlated with periodontal destruction and also with the presence of pathogenic bacteria species such as *Fretibacterium* and *P. gingivalis.* Whether this is due to more severe SLE in subjects with higher prednisone or a direct effect of the drug is open to question. Reports that corticosteroids have little direct impact on the periodontium would argue for the former [38].

Our study is the first to analyze the bacterial composition of the subgingival microbiota in SLE patients using 16S rRNA gene sequencing. This approach allowed a comprehensive view of the subgingival microbial communities associated with SLE. We found that the bacterial load was one log higher in SLE patients with periodontitis compared to control subjects with periodontitis. Alterations in the inflammatory status of the periodontium induced by SLE may impact the subgingival bacterial composition, which in turn may further enhance inflammatory changes and mediate tissue destruction in SLE patients [39]. Consistently higher levels of IL-6, IL-17, and IL-33 were detected in SLE patients with periodontitis. These cytokines are increased in the serum of SLE patients [39–43] and non-SLE individuals with inflamed gingival tissues [44–47]. IL-33 exacerbates periodontal disease [47], increases IL-6 expression [48], and enhances a Th17 response [42]. IL-6 promotes activation and differentiation of T cells, B cells, macrophages, neutrophils, and osteoclasts and promotes formation of Th17 cells [49]. The levels of IL-6, IL-17, and IL-33 were positively correlated with the relative abundance of pathogenic bacteria such as *Selenomonas*, *P. denticola*, *Veilonella*, and *Leptotrichia.* Accordingly, increased inflammation may provide a source of nutrients in the form of tissue breakdown products and alter the redox environment favoring the growth of anaerobic bacteria [3]. In turn, changes in the microbiota might be important to amplify local inflammation and periodontal tissue damage.

SLE influenced microbial diversity, as patients with SLE had lower diversity in sites with periodontal breakdown compared to equivalent sites in matched control subjects. It is possible that the lower diversity facilitates microbial changes that increase susceptibility to periodontitis based on findings of decreased bacterial diversity in inflammatory diseases such as psoriatic arthritis [50] and Crohn’s disease [51]. We also found that bacteria commonly found in periodontal healthy, such as *Capnocytophaga* [52–56], *Rothia* [54, 56–58], *H. parainflunzae* [56, 59], and *Streptococcus* [60] were reduced in proportional abundance in SLE patients with periodontitis, and bacterial species frequently detected in periodontal disease were observed in higher proportions in SLE patients, even in periodontal healthy sites (i.e., *Prevotella oulorum*, *P. nigrescens*, *P. oris*, *S. noxia*, and *Leptotrichia*). In addition, bacterial samples from diseased sites in subjects with SLE were more similar to each other than bacterial samples from healthy sites. Shi et al. [61] also found that bacteria from periodontal disease sites were more similar to each other than bacteria from healthy sites. This was observed in different sites within an individual and in comparison to sites between individuals. These findings suggest that the ecological environment is more consistent and similar in disease than in healthy conditions.

The subgingival microbial community in the SLE patients differs not only in the taxonomic composition but also in the co-occurrence patterns of periodontal microorganisms. Co-occurrence analysis indicates that in the SLE patients, the presence of pathogenic bacteria was positively correlated with other pathogenic bacteria (*T. denticola*, *P. gingivalis*, *T. forsythia*, and *F. fastidiosum)* [32]*.* Shi et al. similarly reported that periodontitis-associated microorganisms were highly correlated in the diseased state but poorly correlated after treatment, suggesting that there are coordinated interactions among the pathogenic microorganisms. It is possible that SLE favors bacterial interactions that lead to increased periodontal disease.

Periodontal disease in SLE patients shows distinct features compared to systemic healthy subjects. SLE may disrupt the balance between host and microbiota in favor of a dysbiotic condition, resulting in increased periodontal damage or an enhanced inflammatory response leading to an increased risk of periodontitis. It is also possible that periodontitis aggravates SLE disease severity, as a worsened periodontal condition was correlated with increased systemic inflammation in subjects with SLE. This result is consistent with reports that periodontal treatment improves clinical outcomes in rheumatic diseases such as rheumatoid arthritis and SLE [16, 26, 62, 63]. Moreover, increases in the proportion of some subgingival bacteria such as *P. nigrescens* has been related to preterm birth [64], rheumatoid arthritis [9, 65, 66], and atherosclerosis [67]. In our study *P. nigrescens* was correlated to higher values of SLE index (SLEDAI and SLICC/ACR), *P. denticola* with increased neutrophil levels, and *Prevotella melaninogenica* with increased serum CRP levels in SLE patients. Other studies in rheumatoid arthritis have found that *Prevotella* species [52, 68] and *A. geminatus* [52] were found in higher levels in RA patients than in healthy controls. These results suggest a possible two-way cycle in which systemic inflammation enhanced by SLE induces dysbiosis of the subgingival microbiota and progression to periodontitis. Periodontitis may then affect the systemic immune response, leading to increased activity of SLE [69].

The present study represents the first comprehensive evaluation of the subgingival microbiota associated with periodontal status in SLE patients and provides a strong basis for further study on the relationship between the two diseases. As a cross-sectional study, the present work has limitations as it is not able to define cause and effect relationship. A longitudinal study in which the same individual was followed just after SLE diagnosis could provide evidence for a cause and effect relationship between the two diseases. This would be particularly useful in identifying bacterial changes associated with the onset of periodontal disease in SLE patients. An intervention trial in which patients are treated for periodontitis would further enhance our understanding as to whether periodontitis has an impact of SLE disease severity.

## Conclusions

SLE patients exhibit a higher prevalence of periodontal disease and increased periodontal disease severity. SLE patients had increased local inflammation and an altered subgingival microbiota, which may account, in part for the periodontal changes. Periodontal inflammation was also associated with more severe SLE scores. The results point to the need to closely monitor the periodontal health of subjects with SLE and for periodontal treatment at early stages, including the removal of subgingival dental plaque.

## Methods

### Subjects

Three hundred thirty-six patients diagnosed with SLE (according to ACR 1982/1997 revised classification criteria) from Rheumatology Outpatient Clinic of Clinics Hospital of Federal University of Minas Gerais (UFMG), Belo Horizonte, Brazil, were initially evaluated. Based on a power analysis at alpha=1 and 5%, 50 subjects will reach a power of 99% when comparing bacterial taxa frequencies between subgingival samples as reported by La Rosa et al. [70]. Fifty-two patients were included in the study after matched the inclusion criteria: Subjects included were at least 18 years of age, no other rheumatic diseases (except for secondary Sjögren syndrome), no treatment for periodontal disease within the last 6 months, no use of orthodontic appliances, no use of antibiotics within the last 3 months, no need for antibiotics for infective endocarditis prophylaxis during dental procedures, no chronic renal insufficiency requiring dialysis or after kidney transplantation, pregnancy or lactation, no acute or chronic infectious conditions at the time of the study visit, no diagnosis of neoplastic disease within the last 5 years, and the presence of at least eight teeth. Additionally, we have excluded from the study patients that did not agree to participate, did not understand the study, or decided to leave the study after the first clinic visit. SLE group was matched for age and gender with 52 subjects without SLE or other known rheumatic diseases (control group).

Medical records were collected for SLE parameters, Systemic Lupus Erythematosus Disease Activity Index 2000 (SLEDAI 2 K) measured SLE activity [71] and Systemic Lupus International Collaborating Clinics/American College of Rheumatology Damage Index (SLICC/ACR) evaluated SLE-associated damage [72]. Periodontal status was assessed by two calibrated examiners. The following parameters were recorded: plaque index, probing depth (PD), clinical attachment level (CAL), and bleeding on probing (BOP). Periodontitis was defined as the presence of two or more interproximal sites with probing depth ≥4 mm or one site with probing depth ≥5 mm [73].

### Sample collection

Subgingival dental plaque samples were collected as described elsewhere [14]. Sterile endodontic paper points (ISO40) (Tanariman, Manacaparu, AM, Brazil) were inserted in the five sites with deepest periodontal pockets and kept there for 1 min. All paper points with subgingival plaque samples were pooled together. After removal, the material was stored in a sterile tube containing 500 μL of sterile distilled water and centrifuged at ×3000*g* for 5 min. The paper points were discharged, and the pellet was kept at −80 °C until DNA extraction.

For saliva collection, patients could not eat or drink an hour before collection procedure. They washed the mouth with pure water and swallowed whole saliva before collection. Saliva was collected by continuous drool into a sterile 50 mL tube for 5 min. The non-stimulated salivary flow was measured in milliliters per minute (ml/min). The saliva samples were subsequently diluted (1:1) in a phosphate-buffered saline (PBS) solution containing protease inhibitors and subsequently frozen at −80 °C until analysis.

Blood samples were collected using a vacutainer with EDTA and immediately placed on ice, clarified by centrifugation at ×3000*g* for 5 min at 4 °C, and kept frozen at −80 °C until assayed.

### DNA extraction and sequencing

Genomic DNA was extract from the samples using the Quick-gDNA MicroPrep kit (Zymo Research, Irvine, CA, USA) and 50 μL (10 mg/ml) of lysozyme per sample for maximal bacterial cell lysis. All procedures were completed in a laminar flow hood with RNase free materials. The quantity and quality of DNA was measured using a spectrophotometer method (Tecan, Männedorf, Switzerland).

The primers 515 F (5′-GTGCCAGCMGCCGCGGTAA-3′) and 806R (5′-GGACTACHVGGGTWTCTAAT −3′) which target the hypervariable V4 region of the 16S rRNA gene were used for amplification [74] followed by gel purification and ethanol precipitation to remove any remaining contaminants and PCR artifacts and subjected to Illumina MiSeq Plataform at the Next-Generation Sequencing Core of University of Pennsylvania.

### Microbiological analysis

Taxonomy was assigned using “Quantitative Insights Into Microbial Ecology” (QIIME) software package [75] with UCLUST against CORE [76]. Alpha rarefaction was performed using the Observed Species and Chao1 metrics. Beta diversity was calculated using UniFrac. Quantification of total bacterial load was determined by real-time PCR using universal primers for 16S rRNA gene (F:AGAGTTTGATCCTGGCTCAG; R: ACGGCTACCTTGTTACGACTT) (IDT, Coralville, Iowa, USA).

### Cytokines measurement

Analyses of IL-6 and IL-17 cytokines were determined using a BD CBA Human Th1/Th2/Th17 Cytokine Kit (Becton, Dickinson and Company, BD Biosciences, San Diego, CA) and analyzed on a BD FACSCalibur flow cytometer (Becton, Dickinson and Company). The concentration of the cytokine IL-33 were measured by enzyme-linked immunosorbent assay (ELISA) using commercially available kits (R&D Systems, Minneapolis, MN, USA). The assays were performed according to the manufacturer’s instructions. The results were expressed as picograms of cytokines and adjusted accordingly salivary flux for salivary samples.

### Statistics

Clinical, demographic, alpha diversity, and bacterial load data were compared using Kruskal-Wallis test with Dunn’s post hoc test. PERMANOVA was performed to compare beta diversity. Correlations between relative abundance of taxa and clinical parameters of periodontal disease and SLE were calculated using Spearman correlation coefficients. For co-occurrence analysis Spearman rank correlation was calculated with cutoff value of rho ≥0.4 and *p* < 0.05 as previously described [77]. Relative abundances of Operation Taxonomy Units (OTU) were compared among SLE and control subjects and tested for statistical significance using DESeq2 [78] and LEfSe [31]. Briefly, LEfSe is a metagenomic biomarker-discovery approach based on an algorithm that first performs a nonparametric Kruskal-Wallis test in order to identify bacterial taxa whose relative abundance is significantly different in a group of interest compared to controls. Subsequently, LEfSe applies linear discriminant analysis (LDA) to those bacterial taxa identified as significantly different (*p* < 0.05) and further assesses the effect size of each differentially abundant taxon. Only those taxa that obtain a log LDA score 2 are ultimately considered. As a result, LEfSe indicates those taxa and OTUs that better discriminate groups. *P* values <0.05 were considered to indicate statistical significance.

## Additional files

Additional file 1: Table S1. Levels of inflammatory cytokines in serum of control subjects and SLE patients. (XLSX 9 kb)
Additional file 2: Table S2. OTU table. (TXT 278 kb)

## Abbreviations

BOP: Bleeding upon probing
CP: Chronic periodontitis
CRP: C-reactive protein
IL: Interleukin
LDA: Linear discriminant analysis
NCP: Non-periodontitis
RA: Rheumatoid arthritis
SLE: Systemic lupus erythematosus
SLEDAI: Systemic Lupus Erythematosus Disease Activity Index
SLICC/ACR: Systemic Lupus International Collaborating Clinics/American College of Rheumatology Damage Index
